# Uncertainties about accepting care robots

**DOI:** 10.3389/fdgth.2023.1092974

**Published:** 2023-05-18

**Authors:** Tuuli Turja

**Affiliations:** Faculty of Social Sciences, Tampere University, Tampere, Finland

**Keywords:** care robot, care robot acceptance, robot acceptability, change management, social representation, telecare, care work, working life

## Abstract

In the midst of the anticipation of care robots renewing elderly care, care workers are expected to orient themselves in this future, however uncertain. To examine how uncertainty over the appropriateness of care-robot use associates with robot acceptance, different scenarios of robot assistance were presented to a sample of care professionals in two waves 2016–2020. The views of usefulness of robot assistance yielded underlying structures of plausible and implausible care-robot use. The perceived appropriateness of utilizing robots in care was stronger in the plausible robot scenarios. The uncertainty about robots having an appropriate role in care work correlated negatively with the perceived usefulness of robot assistance, but was even highlighted among the scenarios of implausible tasks. Findings further show how uncertainties about care-robot use have been reduced across four years between data collections. In robotizing care work processes, it may be more beneficial to attempt to convince the care workers who are undecided about robot acceptance than to push care-robot orientation to those who strongly oppose care-robot use.

## 1. Introduction

By reducing uncertainties about *currently* used technology, the *future* adoption of new and robotic technologies can be efficiently promoted. Perceived uncertainty toward technology has been found to have a significant role in service robot adoption intention in various types of services (1). This brief research report presents a correlative study on care robots and partial evidence supporting the uncertainty hypothesis where uncertainty and acceptance toward robot use is dependent on the type of service.

Care robots are categorized distinct from medical robotics and have been designed to improve the quality of life among, say, elderly people receiving home care (2). It is anticipated that robots, via enhanced artificial intelligence (AI), will change and improve care in the future. However, in order to orient to a care-robot future, care professionals have expressed the need to be better supported and educated (2–4). Care-robot orientation is also put to the test for its fundamental problem of associating care with robots. If care is still defined as *meeting the needs of one person by another person* (5), it will surely rule out robots (6). How plausible is care-robot use from the perspective of those who are committed to the care needs of patients?

The first objective of this study was to look into future telecare robot scenarios presented to professional care workers. As scenarios differed from each other in type of service (1), they were presumed to have the potential to be perceived as either plausible or implausible outlooks for care robots (7). Second, the scenarios were examined in association with expressed uncertainty regarding care-robot use.

RQ1: Do robot assistance scenarios yield separate structures implying plausible and implausible futures of robot use?

RQ2: How does uncertainty regarding the appropriateness of care-robot use associate with robot acceptance in both plausible and implausible care-robot scenarios?

The acceptance of robots among individuals has been a growing topic of research. However, uncertainty toward robots among working populations has received less attention (1). The current study used survey data on Finnish elderly-care workers' perceptions and acceptance of robots, collected during November–December 2016 and November–December 2020. For this study, Finland made a promising example of a society that is ready to invest in new technologies in welfare services (8), but also has national strategies to prioritize healthcare and social workers' needs and preferences in digitalization (9). Research is needed because perceptions and social representations of robots are culturally and contextually dependent (10). Technological orientation, as a wider concept, has also been shown to vary between populations (11).

## 2. Background

Already technologized healthcare (12,13) is being transformed by new and intelligent service technologies. Some of these new technologies appear as entirely new for their users and are in greater need of becoming accustomed to. Other new technologies are more familiar since they are a result of development in which a certain type of technology is first implemented in one context and later applied in others. In these latter cases, new technologies are easier to appreciate because they require less imagination before understanding their value in everyday life. Even with novel types of technologies, people rely on their current knowledge. In a situation in which robots are brought into a new user context, existing social representations guide people to mentally anchor novel technologies to already familiar technologies (10).

Examples of familiar but evolving technologies are virtual assistants and advanced chatbots (e.g., ChatGPT) that we are used to having on our mobile devices but can also be applied in a care context. In the context of home care, these AI assistants have the potential to operate, not only to provide reminders for medications or appointments, but also as conversational entities that can offer advice and self-care recommendations (14,15). Even if integrated into a robotic device, the basic concept of a virtual assistant remains the same and is thus easy to anchor to previous knowledge about assistive technologies (10). Nevertheless, when virtual assistant software is integrated into a hardware robot, it must be categorized again—this time, as an embodied artificially intelligent assistant (16).

The most familiar types of robots in elderly care are medicine-dispensing robots typically used in home care and robotic pets typically used in care facilities (17). Other types of robots used in a care context today include social robots (e.g., the humanoid robot Pepper) and telepresence robots developed to mediate mobile robot-mediated human–human interaction. In care homes where many residents suffer from dementia and other cognitive impairments, access control is an important safety issue. Access control technology includes, for example, wearable bracelets, which are problematic because they tend to rely on the user's compliance (18). Access control delegated to a robot would be embodied by a robotic system that detects environmental changes, identifies people and their access rights, and reacts appropriately to the observations it makes.

This brings us to the importance of culturally and contextually appropriate robot assistance. Without considering the social norms and appropriateness of implementing robots in a novel context, any organization would be at risk of failing to implement a responsible technological change (19). In some cases, organizational changes demand that their employees follow suit and refrain from any criticism of the change. In a worst-case scenario, this means expecting employees to abandon their personal principles or even attempt to breach occupational ethics in the name of keeping up with global innovations (17, 20).

Among the novel fields to be robotized, care-work robots form a particularly sensitive subject. Care robotization entails concerns about replacing human work and human contact with a machine, as well as values of care with robotic logic (6). The value-based considerations can raise uncertainties regarding the mere idea of using robots in care. Uncertainty theory explains how various contextual features affect the certainty in which individuals are able to see different futures as plausible (1). In addition to values of care, uncertainties toward care robots can originate from the implausible depictions of autonomous and human-like robots illustrated in the media (21). Firsthand experiences of care robots as simple preprogrammed machines are not always in line with the exaggerations of robots shown in the media. The narrative of advanced multifunctional care robots can increase the fear of losing work to new technologies, as well as bring to the surface any dystopian ideas of healthcare run by robots. Social representations affect robot acceptance on many levels, both practical and principled.

Perceived usefulness is one of the key factors in care-robot acceptance, where robot use is being measured for its potential to improve work and the roles of the human employees (22–25). However, differentiating from this tradition of user experience (UX) studies, perceived usefulness of robots is viewed here as the hypothetical usefulness of robot assistance in different future scenarios. Because the perceptions of robots and their imagined usefulness in care work are highly contextual, this study divided the concept of robot assistance into separate scenarios of different types of services. According to Wiek et al., uncertain future scenarios can be measured by their plausibility (7). In this method, scenarios can be presented as the most reasonable futures possible, but they can also be built more provocatively to deliver a comparison setting (26) or to bring about also more unpredictable responses (27).

**Figure 1:**
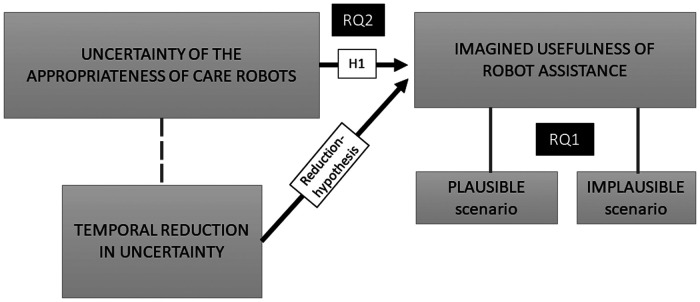
Study design.

On the basis of Liu et al.'s (1) conclusion that different contextual scenarios of robot assistance influence a potential user's evaluations of uncertainty and acceptance of robots, it is first hypothesized that the robots' appropriateness in care is perceived greater among the plausible robot use scenarios. Next, it is hypothesized that temporally reduced undecidedness about robots' appropriateness in care is associated with higher appraisals of care-robots' usefulness. The reduction-hypothesis is tested using a subsample of care workers who reported more uncertainty toward care-robot use in 2016 than they did in 2020. The study design is presented in Figure 1.

## 3. Method

This study of elderly-care workers' views toward care robots is based on online survey data from 2016 (T1) and 2020 (T2). For T1, participants were randomly sampled from the member registers of the Finnish Union of Practical Nurses and the Union of Health and Social Care Professionals in Finland (*N* = 3,800). At T2, the survey was repeated with the 426 participants from T1 who registered for a follow-up study in 2016. The response rate of 56% for T2 produced a sample of 238 respondents.

The respondents were mostly female (94%) and ranged from 24 to 67 years old at the time of the second data collection (*M* = 50.50, *SD* = 11.30). Occupations included practical nurses (54%), registered nurses (27%), and miscellaneous occupational groups (19%). In 2020, the majority of the respondents (57%) did not have any experience working with care robots. Responding to the online questionnaire, the respondents were introduced to robots using written definitions and illustrations.

Statistics are reported as percentages, means (*M*), standard deviations (*SD*), and Pearson correlation coefficients (*r*). Comparisons between dependent samples used the nonparametric Wilcoxon signed-rank test (Z). Dependent samples refer here to, both, the cross-sectional setting where the respondents evaluated repeated scenarios, and to the longitudinal setting between two measuring points.

### 3.1. Variables

The appropriateness of robot assistance was operationalized as the statement, “robots are meant to be used in care work,” which was modified for specific reference to robots from the value-based technology acceptance questionnaire (22). The response scale ranged from 1 to 5: *totally agree, somewhat agree, neither agree nor disagree, somewhat disagree, and totally disagree*. In 2016, the responses were on the negative side (*M* = 3.33; *SD* = 1.23), whereas in 2020, the responses were on the positive side (*M* = 2.25; *SD* = 1.13).

**Figure 2:**
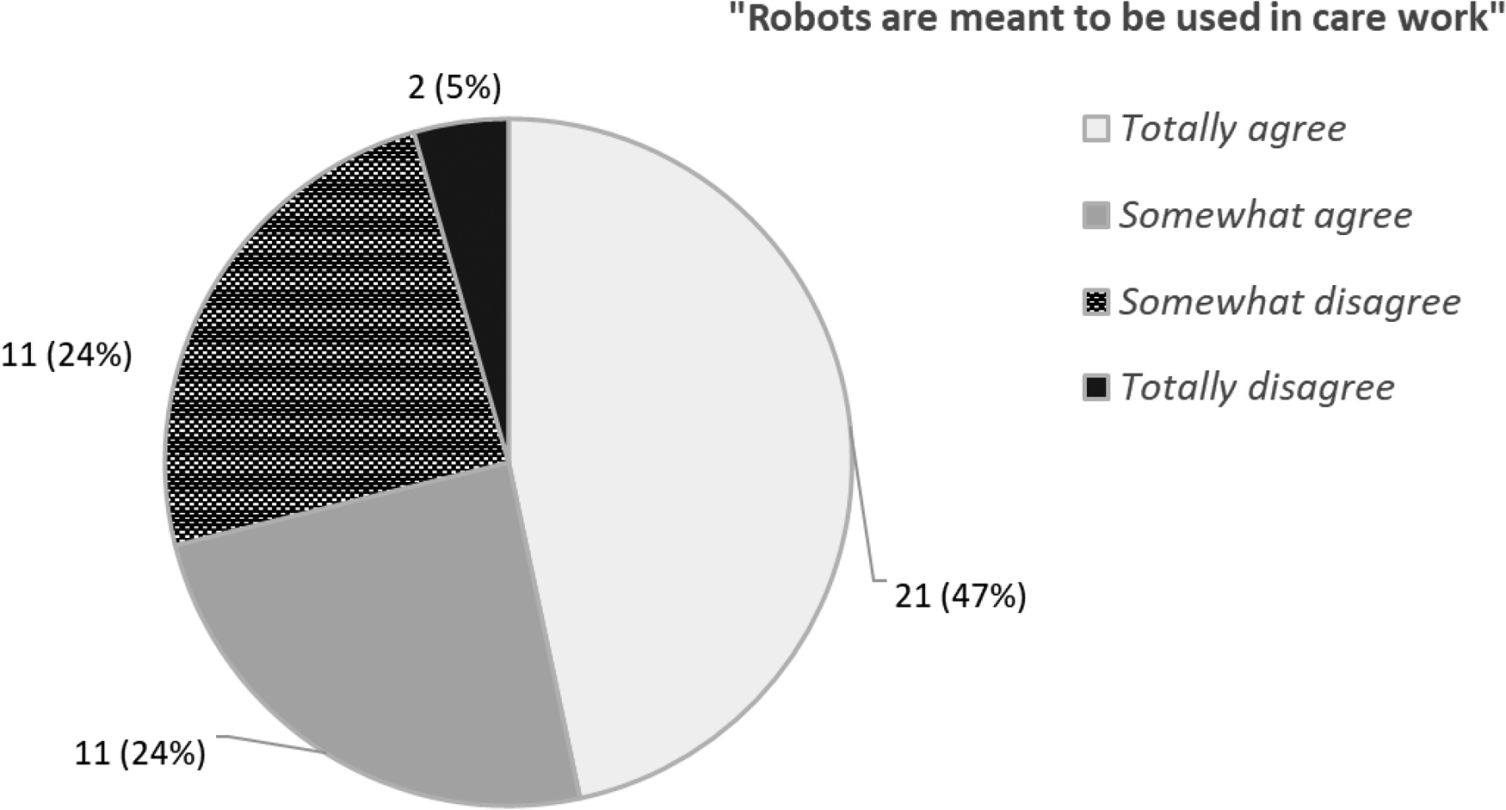
Reduced undecidedness: responses (*n *= 45) indicating undecidedness changed from 2016 to a stronger viewpoint in 2020.

One of the drivers for setting the research questions in this study was the uncertain responses found among the responses on the appropriateness of robot assistance. In T1, a notably large proportion of the respondents (21.6%) answered that they neither agreed nor disagreed with the statement, while in T2, the proportion of the undecided had decreased by about half (11.7%). The uncertain responses in T1 and T2 were first categorized on the grounds of which year of data collection they had given the response “neither agree nor disagree” and the direction of the possible temporal change. The longitudinal data included 67 respondents who had been undecided on the appropriateness of robot assistance at either T1 or T2. Only four of the respondents had been undecided during both T1 and T2. About one-quarter of the respondents (18) had a stronger viewpoint in 2016 than in 2020. Thus, the largest group included the 45 (67%) respondents who were undecided only in 2016 and had formed a stronger viewpoint by 2020. In order to test the hypothesis, the analysis focused on the latter group. The distribution of these responses is shown in Figure 2.

**Figure 3:**
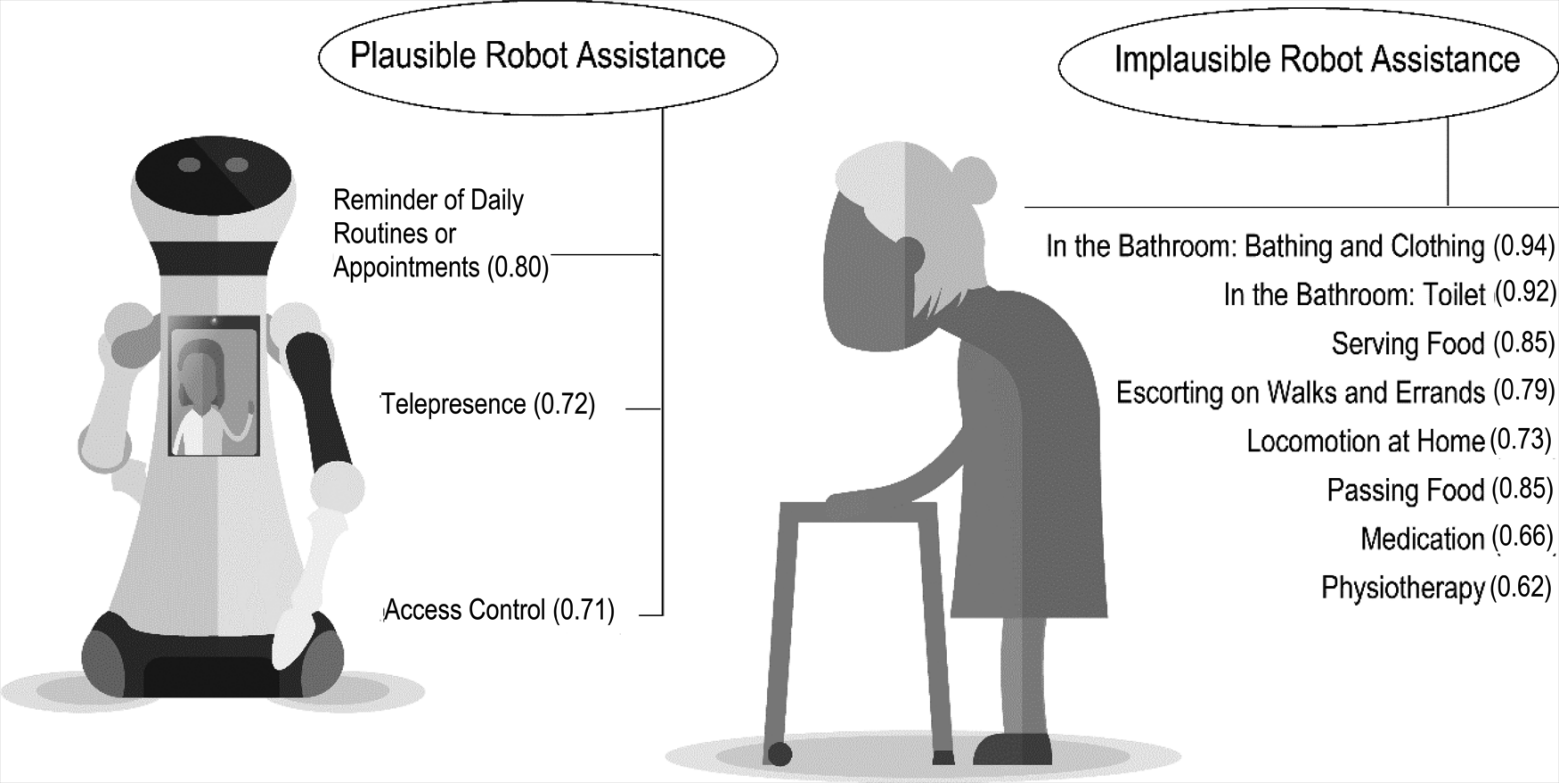
Scenarios of robot assistance: principle components analysis, pattern matrix.

The perceived usefulness of care robots was measured in the questionnaire using 12 scenarios presenting robot assistance as task-specific telecare solutions (Figure 3). Scenarios were independent from each other, meaning that the respondents did not have to decide or prioritize between them, but evaluate each scenario on its own (6). Evaluations were given on a scale from 1 to 10 for the question “How would you consider telecare robots’ usefulness in different tasks?,” with 1 being the least useful and 10 being the most useful. Measures of perceived usefulness in this study were utilized in a cross-sectional setting using only data from T2. An average perceived usefulness in 2020 implied a relatively high level of robot acceptance (*M* = 6.84, *SD* = 1.97, *α* = 0.91). These statistics have been reported in a previously published research article [Author A, anonymized], but the perceived usefulness has not been attempted to be categorized into separate underlying structures before.

The first research task was to reduce the number of variables by making combinations of the scenarios. A cross-sectional principal components analysis (PCA) utilized the T2 dataset and its interrelated scenarios. PCA was conducted to identify underlying structures in the perceived usefulness of care robots, and more specifically, to produce information about scenarios that load similarly regarding their evaluated *plausibility*. This approach is distinct from the research tradition of future scenarios that focus on the *likelihood* of different outlooks (28). PCA was conducted using oblique rotation. A direct oblimin estimation was chosen because of the high correlation among the evaluations of the scenarios. The T2 data showed good fitness for PCA. The Kaiser–Meyer–Olkin test implied a satisfactory sample size (KMO = 0.88), and Bartlett's test of sphericity suggested an adequate number of correlations between the variables (*p* < 0.001). During the analysis, one item loaded on more than one component and was removed, after which the analysis was re-run. Thus, the number of scenarios used in the final analysis was reduced to eleven.

## 4. Results

Appropriateness of robot use received more positive appraisals from care workers in T2 than it did four years prior in T1 (Z = 7.68; *p* < 0.001). Undecidedness in the responses was also alleviated between the two periods. A clear majority (71%) of the respondents who had been uncertain about the appropriateness of using robots in care in 2016 had formed a robot-accepting viewpoint by 2020 (Figure 2).

Eleven scenarios relating to the perceived usefulness of care-robot use were analyzed using PCA, looking for structural similarities. Parallel analysis as the preliminary step for PCA (29), indicated two components to retain from the data. The loadings are presented in Figure 3. The first category included three items (*M* = 8.16; *SD* = 1.85; *α* = 0.69), and the second category included eight items (*M* = 6.31; *SD* = 2.36; *α* = 0.93). Statistically, the two components differed from each other in terms of the shared variance of the variables included. The components were interpreted to differ depending on the perceived plausibility of the scenarios.

Both a scree plot and produced eigenvalues supported the two-component structure. The two categories deriving from the imagined usefulness of robot use explained a total of 68.17% of the variance in the data. Component 1 was labelled as plausible robot assistance and explained 16.61% of the variance. Component 2 was labelled as implausible robot assistance and explained 51.57% of the variance. The component loadings for plausible and implausible robot assistance are presented in Figure 3 as an illustration of the pattern matrix. Scenario of a robotic reminder emerged as the most important loading for plausible robot assistance. Scenario of bathing and clothing was found the most important loading for implausible robot assistance.

Supporting the first hypothesis, robot assistance in the plausible scenarios was perceived as more useful (*M* = 8.16) than robot assistance in the implausible scenarios (*M* = 6.31; Z = 11.67; *p* < 0.001). In both the datasets of T1 and T2, the care workers who were uncertain of whether robots could possess an appropriate role in care work were prone to find robots less useful in different care-work tasks. In T1, slight but statistically significant negative correlations were found between uncertainty over the appropriateness and perceived usefulness of care robots (*r* = −0.140; *p* < 0.001) and its subcategories, the perceived usefulness of plausible robot assistance (*r* = −0.100; *p* < 0.001) and the perceived usefulness of implausible robot assistance (*r* = −0.128; *p* < 0.001). Similarly, in T2, correlations were found between uncertainty and the perceived usefulness of robot assistance (*r* = −0.140; *p* < 0.05) and its subcategory, the usefulness of implausible robot assistance (*r* = −0.134; *p* = 0.05).

The factor scores calculated for plausible and implausible robot assistance were used to test the hypothesis of reduced uncertainty enhancing robot acceptance. However, the temporal change in undecided responses did not have enough statistical power to explain the variance in plausible and implausible robot assistance.

## 5. Discussion

Although not a conventional approach to focus on the undecided responses to a questionnaire, the uncertainty model (1) offered a theoretical tool to examine the staggering number of the responses “neither agree nor disagree” to an item where elderly-care workers were asked about their views on the appropriateness of care-robot use. In line with Liu et al. (1), this study shows how undecidedness correlates with service robot acceptance−also in a care context. Although the specific reduction-hypothesis was rejected, the cross-sectional results showed in this study that uncertainty does play a part in robot acceptance, both in plausible and implausible scenarios. To be able to reduce these uncertainties among the potential users is a step toward more approving robot adoption. When promoting care-robot orientation, rather than convincing people resistant to care robots, it may be more beneficial to attempt to win over those who are still undecided about robots.

Indeed, between the years of data collection, uncertainty about robots having an appropriate role in care work correlated consistently with a lesser perceived usefulness of care robots. Particularly, implausible and more futuristic scenarios seem to be approached with skepticism by the care workers who are uncertain about the appropriateness of using robots in care. The findings of plausible robot assistance receiving more accepting appraisals than implausible scenarios supports our hypothesis (H1) and the theories in which subjective viewpoints are understood as being strongly shaped by our mental and social representations (10, 30).

Exceptionally high component loadings in plausible and implausible robot assistance indicate coherence of the model, and further yet, provide a promising incentive for theory building. There is also face validity in the found dichotomy of plausible and implausible robot assistance. Scenarios of robot assistance in the bathroom loaded strongly on the principal component of implausible robot assistance, where robots are not easily considered useful telecare solutions. Imagining care-robot use in such sensitive, intimate, and fine-motoric tasks seems to take effort. On the other hand, medication and physiotherapy are less significantly on the implausible side of robot assistance. This can be explained by the relatively evident anchors between already known telecare systems and imagined robotic physiotherapy and medication assistance.

Telepresence, access-control, and personal-assistant robots were service types included in plausible scenarios. Computer-to-computer calls are widely used in both working and private life. The worldwide Covid-19 pandemic further accelerated the use of video-based telecommunication, normalized teleconferences, and found new contexts for utilizing computer-to-computer calls. Thus, telepresence robots as video-call devices on wheels are perceived as a plausible type of technology for renewing care work because they anchor explicitly to an already familiar type of technology (10). Similarly, access-control robots anchor to already known technologies (19) and can be viewed as particularly useful in a time of data collection in which elderly care had struggled with the pandemic and social distancing for a year. The third plausible scenario included robotic assistance in schedule reminders, which has an evident anchor to virtual assistants that people use on mobile devices. Thus, a chatbot or virtual assistant software integrated into a robot (16) is viewed as holding enough credibility to qualify as possible (7).

As a limitation of this study, the longitudinal data could not be properly utilized. The analysis testing the hypothesis of the temporal change in uncertainties over robot-use appropriateness and its relation to the perceived usefulness of care robots did not hold statistical significance. The findings are therefore limited to associations that emerged from both of the measuring points separately. The sample size, *per se*, was considered reasonable for the cross-sectional PCA, where the number of respondents was 21 times larger than the number of variables (31). Furthermore, although the loadings between the constructed principal components were not even, the structure of plausible robot assistance included three variables, which is usually considered to reach the minimum number of acceptable loadings (32).

Future service robot studies should consider and advance the theory of plausible and implausible robot assistance. This work follows a service robot study that emphasizes the role of various scenarios as a tool to deepen the understanding of the viewpoints of potential robot users (1). Scenarios are acknowledged as a recommendable methodology in the attempts to learn which futures of robot use are riddled with the most uncertainty and which, on the contrary, are viewed both as preferable and plausible.

In summary, this empirical study provides new information about care workers' preferences for robots and preliminary knowledge of the undecidedness that comes with those preferences. Robotic scenarios that are perceived as implausible associate with less acceptance and more uncertainties. In the objective of creating more acceptable views of care robots among the staff, employers should be aware of how the current opinions and attitudes are formed and how they can be affected. Fears and uncertainties can often be alleviated by providing a reasonable amount of reliable information.

## Data Availability

The original contributions presented in the study are included in the article, further inquiries can be directed to the corresponding author/s.
